# Numerical and Non-numerical Predictors of First Graders’ Number-Line Estimation Ability

**DOI:** 10.3389/fpsyg.2018.02336

**Published:** 2018-11-30

**Authors:** Richard J. Daker, Ian M. Lyons

**Affiliations:** Department of Psychology, Georgetown University, Washington, DC, United States

**Keywords:** number-line estimation, spatial processing, early numeracy, gender differences, number symbols

## Abstract

Children’s ability to map numbers into a spatial context has been shown to be a powerful predictor of math performance. Here, we investigate how three types of cognitive abilities – approximate number processing ability, symbolic number processing ability, and non-numerical cognitive abilities – predict 0–100 number-line estimation performance in first graders. While each type of measure predicts number-line performance when considered individually, when considered together, only symbolic number comparison and non-verbal reasoning predicted unique variance in number-line estimation. Moreover, the relation between symbolic number comparison and number-line ability was stronger for male students than for female students, suggesting potential gender differences in the way boys and girls accomplish mapping numbers into space. These results suggest that number-line estimation ability is largely reflective of the precision with which symbolic magnitudes are represented (at least among boys). Our findings therefore suggest that promoting children’s understanding of symbolic, rather than non-symbolic, numerical magnitudes may help children learn better from number-lines in the classroom.

## Introduction

Children’s ability to map numbers into a spatial context has been shown to be a powerful predictor of math performance (Siegler and Booth, 2004; Booth and Siegler, 2008; Sasanguie et al., 2013; Lyons et al., 2014; Friso-van den Bos et al., 2015; Schneider et al., 2018). Past research using number-line estimation tasks, in which children mark the spatial location of a given number (e.g., “72”) on a horizontal line (typically with only the endpoints indicated, e.g., with 0 at the left end and 100 at the right end), has been shown to predict performance on other measures of basic numeracy (Laski and Siegler, 2007; Maertens et al., 2016) and arithmetic (Siegler and Booth, 2004; Booth and Siegler, 2008; Lyons et al., 2014; Schneider et al., 2018). Moreover, experimental research has demonstrated that playing board games meant to bolster the visuospatial representation of numerical values in children improves numerical knowledge and performance on a range of numerical and mathematical tasks (Ramani and Siegler, 2008; Siegler and Ramani, 2009; Ramani et al., 2012; Maertens et al., 2016). The precision with which children perform number-line estimation tasks has been argued to reflect the precision with which children represent numerical magnitudes (Laski and Siegler, 2007; Booth and Siegler, 2008), which has been proposed by some researchers to serve as a key foundation for more complex mathematical processing (e.g., Feigenson et al., 2013; Siegler and Braithwaite, 2017). Given both the predictive and potentially causal role that visuospatial representations of numerical magnitude play in the development of mathematics, an important question is what basic numerical abilities contribute to the early development of these visuospatial representations.

Past work examining number-line estimation ability has in part focused on pinpointing when key developmental shifts occur (Siegler and Opfer, 2003; Siegler and Booth, 2004; Booth and Siegler, 2008; Siegler and Ramani, 2009). Of particular importance, multiple studies have found that by the time children are in second grade, students have developed a fairly linear 0–100 mental number-line, whereas children in first grade are, on average, still in the process of linearizing their visuospatial representations of 0–100 (Siegler and Booth, 2004; Booth and Siegler, 2006). The development of this mental number-line has been theorized to have a core role in broader numerical cognition (Siegler et al., 2011, 2013). Siegler et al. (2011) have argued for an integrated theory of numerical development in which numerical development involves coming to understand that “all real numbers have magnitudes that can be ordered and assigned specific locations on number-lines.”

While the development of a precise mental number-line is thought to play an important role in broader numerical development, it is important to note that performance on the number-line task is not a pure reflection of children’s numerical understanding. Recent work has shown that non-numerical factors, particularly strategy selection, play a substantial role in children’s number-line performance (Barth and Paladino, 2011; Cohen and Blanc-Goldhammer, 2011; Slusser et al., 2013; Rouder and Geary, 2014; Dackermann et al., 2015; Peeters et al., 2016, 2017; van’t Noordende et al., 2016). The role that individual differences in strategy selection play in number-line performance makes it important to consider non-numerical factors, such as non-verbal reasoning ability, that may impact children’s performance on the number-line task.

Given the centrality with which increasing precision of the mental number-line is theorized to play in more general numerical development, understanding what basic numerical and non-numerical cognitive abilities predict the ability to precisely map numbers into space during key developmental shifts can give us insight into possible mechanisms that could underlie core numerical abilities. The goal of the present research is to understand what basic numerical and non-numerical cognitive abilities predict the ability to precisely map numbers into space during a key developmental period.

Here we consider three main hypotheses about what types of basic numerical and non-numerical cognition may support visuospatial number-line estimates in early grade school. According to one view, approximate number processing has been argued to be the foundation upon which more complex numerical abilities are grounded (Dehaene, 1997; Libertus et al., 2011, 2012, 2013; Feigenson et al., 2013). Because number-line estimation abilities are still developing in first graders (Siegler and Booth, 2004; Booth and Siegler, 2006), it may be the case that individual differences in approximate number processing at this age are predictive of number-line abilities. More specifically, this view predicts that a common measure of approximate number processing (i.e., determining which of two arrays of dots contains the greater quantity) should be a robust predictor of number-line estimation accuracy.

A second view is that symbolic representation of numerical quantities (e.g., Indo-Arabic numerals) serves as a crucial conceptual leap that underpins much of the subsequent development of more complex numerical thinking (e.g., De Smedt et al., 2009; Bugden and Ansari, 2011; Merkley and Ansari, 2016; Vanbinst et al., 2016; Núñez, 2017). A canonical measure of basic symbolic number processing is via numeral comparison tasks in which children indicate which of two numerals (e.g., ‘6’ and ‘8’) represents the greater quantity. Performance on this task has been shown to be a strong predictor of math achievement across a wide range of ages and settings (Holloway and Ansari, 2009; Nosworthy et al., 2013; Vanbinst et al., 2016; Sasanguie et al., 2017; Lyons et al., 2018). Moreover, previous work has shown that improvements in number-line estimation accuracy are associated with improvements in numeral comparison ability (Laski and Siegler, 2007; Ramani and Siegler, 2008), indicating that these two basic numerical abilities may be fundamentally intertwined early in development. However, it remains less clear whether these two abilities are *uniquely* related – that is, does the relation obtain even after controlling, for example, for approximate number processing, general cognitive ability, and other basic numerical abilities such as counting, ordering and estimation.

A third hypothesis is that reasoning or general cognitive ability – more so than other basic numerical abilities – is the strongest predictor of number-line estimation in early grade-school. As the work demonstrating effects of strategy utilization shows (e.g., Slusser et al., 2013; Peeters et al., 2016), numerical understanding is not the only thing that contributes to number-line performance. It is therefore possible that children with higher levels of general reasoning ability will demonstrate better number-line performance (even after controlling for basic numerical abilities), via the ability to select the most effective strategies.

Of course, the hypotheses outlined above are not mutually exclusive. Indeed, previous studies have demonstrated that measures of all three kinds significantly relate to number-line performance (Opfer and Siegler, 2007; Sasanguie et al., 2012; Fuhs and McNeil, 2013; Fazio et al., 2014; Maertens et al., 2016). However, to our knowledge, no work has examined the *unique* contributions of these numerical and non-numerical abilities to number-line estimation. Learning what predicts unique variance in number-line estimation ability will allow for a more precise understanding of which aspects of early numeracy are foundational in the development of a precise mental number-line. Such an understanding would allow for the generation of testable hypotheses about how to improve number-line estimation ability (and in turn math skills).

While the measures mentioned above are of primary theoretical interest, assessing the extent to which other basic numerical abilities (i.e., numerical ordering ability or counting proficiency) predicts number-line estimation performance comes with at least two benefits: First, it is possible that the three hypotheses outlined above are incomplete – testing other basic abilities allows us to check for additional factors that may impact number-line estimation performance not covered by those hypotheses. Second, given that other basic numerical abilities have also been shown to predict more complex math (Lyons and Beilock, 2011; Lyons et al., 2014), it is important to control for these other abilities to estimate as precisely and conservatively as possible the unique variance in number-line performance that can be attributed to the measures of primary theoretical interest outlined above.

In this study, we used data from over 200 Dutch first graders to understand what basic numerical and general cognitive factors predict unique variance in 0–100 number-line performance. We chose to focus on first graders because past work has suggested that important developmental shifts in 0–100 number-line performance occur during this year (Siegler and Booth, 2004; Booth and Siegler, 2006), and because this age group shows sufficient variability in terms of individual differences in our sample to allow for meaningful inferences to be drawn from a multiple regression approach. Finally, given substantial evidence for gender differences in number-line estimation, especially in first grade (Thompson and Opfer, 2008; Gunderson et al., 2012; Hutchison et al., 2018), we assess whether the strength of the potential relations between basic numerical and non-numerical cognitive abilities and number-line estimation depends on (i.e., interacts with) gender.

## Materials and Methods

### Participants

235 Dutch children (105 female; mean age = 7.06 years; *SD* age = 0.44) in first grade participated. Of this initial sample, 24 were removed from analysis for chance performance on any of the tasks and another 3 were removed for scores on any task that were greater than 4 standard deviations away from the mean. Of the initial sample of 235, 27 were removed (11.5%) for a total analytic sample size of 208 (97 female).

It is important to note that the data reported here are part of a larger data set, some of which has been reported on in previous work (e.g., Lyons et al., 2014). Crucially, both the theoretical questions addressed and the analyses described here are novel.

### Procedure

The ethics review board at Maastricht University approved the data collection procedure used in this study. Children came from seven different primary schools in the Netherlands, where data collection took place. The schools provided written notification of the purpose and nature of the data collection procedures to parents. Parents could withhold consent by returning the appropriate form. All data were collected one-on-one by trained project workers at the children’s schools. All data were collected in one session. All measures were computerized with the exception of the non-verbal intelligence measure (Ravens), which was in a paper-and-pencil format. Before each numerical task, participants were given 3–6 practice trials. During the main experimental trials, no feedback was given for any of the tasks.

### Primary Tasks of Interest

#### Number-Line Estimation (NumLine)

In the NumLine task, children were shown a horizontal line with 0 marked on the left side and 100 marked on the right. On each trial, participants saw an Arabic numeral centered above the line and heard the same number over headphones. Their task was to click where on the number-line the target number should be placed based on the quantity it represented. All stimuli remained on the screen until the child responded. Children completed a total of 26 trials. Reliability on this task was high: alpha = 0.90.

Consistent with previous research on the 0–100 number-line task (Siegler and Booth, 2004; Booth and Siegler, 2006), performance on this task was near ceiling for children above first grade in the broader dataset from which this study is drawn. Ceiling-level performance dramatically reduces variability of scores in older children, making individual-differences-based results with this task in older children largely uninterpretable. On the other hand, we did see substantial variability in performance among first graders; this coupled with the observation that meaningful developmental changes are still occurring on this task in first graders (see Introduction) prompted us to focus on first graders for the purposes of the present research.

### Numeral Comparison (NumComp)

In the NumComp task, children were shown two Arabic numerals presented horizontally, and their task was to decide which number was greater. A total of 64 trials were presented, comprised of 32 one-digit and 32 two-digit trials. Four ratio (*R* = min/max) ranges were used: *R* < = 0.5, *R* = 0.5, 0.5 < *R* < 0.7, and *R* > = 0.7. Each ratio range occurred equally across one- and two-digit trials. All stimuli remained on the screen until the child responded. Reliability on this task was high: alpha = 0.92.

### Dot Comparison (DotComp)

In the DotComp task, children were shown two dot arrays, and their task was to decide which array contained more dots. 64 trials were presented, and quantities and ratios used were identical to those in the NumComp task. Overall area and average individual dot-size were always incongruent with number such that the array with fewer dots always had greater overall area and larger average dot-size. This was done to preclude participants from using strategies based on surface area or dot size to determine which array contained the greater quantity of dots. Additional stimulus details for this task, including manipulation checks, can be found in Lyons et al. (2014). All stimuli remained on the screen until the child responded. Reliability on this task was high: alpha = 0.92.

#### Non-verbal Intelligence (Ravens)

The Ravens task is a normed, timed, visuospatial reasoning test for children (Raven et al., 1995). A colored pattern appeared and children were asked to select the missing piece out of six choices. The task was comprised of a total of 36 trials, and the total number answered correctly was the child’s score. Van Bon (1986) reported reliabilities of 0.80 or higher for the Dutch version of this task.

### Additional Numerical Tasks of Secondary Interest and Covariates

#### Numeral Ordering (NumOrd)

In the NumOrd task, children were shown three single-digit Arabic numerals presented horizontally. On half of the trials, the three numbers were in increasing order from left to right. On the other half of trials, numbers were either in decreased or mixed order. Children were instructed to indicate with a button press whether the numbers were in increasing order or not. All stimuli remained on the screen until the child responded. The 28 trials were roughly divided into distances of 1–3. For example, an in-order trial with distance 1 may contain the numbers “4, 5, and 6” whereas an in-order trial with distance 3 may contain the numbers “2, 5, and 8.” Reliability on this task was high: alpha = 0.82.

#### Object Matching (ObjMatch)

In the ObjMatch task, children were presented with a sample array of common objects (including animals and fruits) and two test arrays. The children’s task was to select the test array that contained the same number of items as the sample array. A total of 45 trials were shown: in 15 trials, all objects in each of the arrays were the same; in 15 trials, each array contained different types of objects (but the objects within an array were of the same type); and in the remaining 15 trials, each array contained a mixture of object types. The number of objects in the arrays ranged from 1 to 6, and the difference in the number of objects between the two test arrays was 1 or 2. All stimuli remained on the screen until the child responded. Reliability on this task was high: alpha = 0.92.

#### Dot Quantity Estimation (DotEst)

In the DotEst task, children saw a single array of dots presented for a very short time (750 ms) – too quickly to be counted individually – followed by a visual mask. The task was to estimate the amount of dots present in the array with a verbal response, which was manually recorded by the experimenter. This task contained a total of 84 trials, made up of 12 trials each with the quantities 1, 2, 3, 4, 7, 11, and 16. Note that results do not substantially change if only quantities 7, 11, and 16 are used. Reliability on this task was acceptable: alpha = 0.76.

#### Counting (Counting)

In the Counting task, children were presented with between 1 and 9 dots, and their task was to count the number of dots as quickly and accurately as possible. This task contained a total of 45 trials, 5 with each quantity. Children responded verbally, and their responses were manually recorded by the experimenter. Children were instructed to press a button as they gave their response in order to estimate response times. Reliability on this task was high: alpha = 0.90.

#### Visual-Audio Matching (VisAud)

In the VisAud task, children heard a number word spoken aloud and were immediately presented with an Arabic number on the screen. The task was to indicate by button press whether the numbers were the same. This task was comprised of 64 trials, half involving one-digit numbers and the other half involving two-digit numbers. On trials in which the numbers did not match, the ratio between the numbers ranged from 0.25 to 0.89. Moreover, non-matching trial stimuli avoided tens-ones confusion items (e.g., 32 and “twenty-three”). Reliability on this task was high: alpha = 0.90.

#### Reading Ability (Reading)

The Reading task was part of the Maastricht Dyslexia Differential Diagnosis battery (Blomert and Vaessen, 2009). Children completed three subtasks that contained high-frequency words, low-frequency words, or pseudo-words. For each subtask, participants were shown up to five screens, each with up to 15 items, for a total of 75 items per subtask. Children were tasked with reading each item aloud as quickly and accurately as possible in 30 s. This task was included to control for basic reading fluency in the multiple regression analyses. The Reading score was the total number of words correctly read across each subtask. Test-retest reliability reported for this task is 0.95 (Blomert and Vaessen, 2009).

#### Basic Stimulus-Response Processing (StimResp)

In the StimResp task, children were presented with four boxes arranged horizontally on the screen. On each trial, a fish appeared in one of the four boxes, and the children’s task was to press the corresponding key on the response box as quickly and accurately as they could. Children completed a total of 20 trials. This task was included to control for basic stimulus-response processing in the multiple regression analyses. All stimuli remained on the screen until the child responded. Reliability on this task was high: alpha = 0.88.

### Task Scoring

For the NumLine and the DotEst task, we used percent absolute errors: *PAE* = | *Est* – *Target*| /*Scale*, where *Est* is the child’s estimate, *Target* is the target number, and *Scale* is the range of target numbers. The range was 100 for the NumLine task and 16 for DotEst. For the NumLine task, note that results were highly similar if degree of linearity (a child’s *R*^2^ indicating the linear fit between their estimates and the actual value) was used instead of PAE. A higher value thus indicates *poorer* performance on these tasks; for this reason, values were multiplied by -1 before being entered into regression models.

For tasks in which error rate and response time data was available (NumComp, DotComp, Counting, NumOrd, VisAud, ObjMatch, and StimResp), we used a composite of error rates and response times on correct trials: *P* = RT(1 + 2ER), where RT is a child’s mean response-time for that task and ER is the child’s error-rate for that task (Lyons et al., 2014). This was done to account for speed-accuracy tradeoffs and to cut down on the number of analyses required, thus minimizing the risk of Type 1 errors. A higher value thus indicates *poorer* performance on these tasks; for this reason, values were multiplied by -1 before being entered into regression models.

We used total number of correct responses for both the Ravens and Reading tasks, hence a higher value indicates *better* performance on these tasks.

## Results

### Basic Descriptives

**FIGURE 1:**
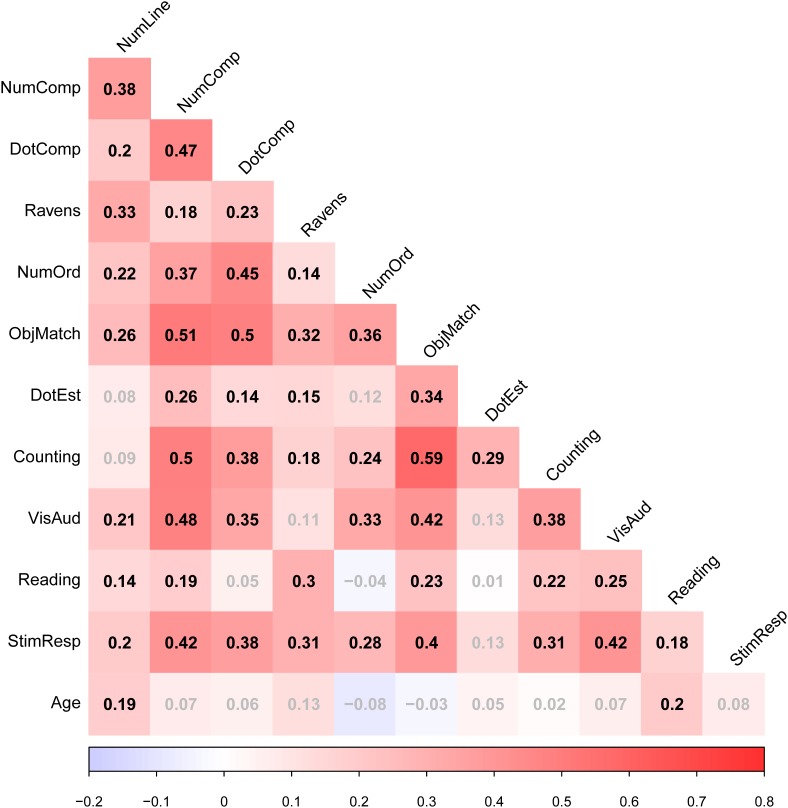
Zero-order correlation matrix. The figure shows zero-order correlations.

Table 1 shows mean performance levels for each task (before multiplying relevant scores by -1), and Figure 1 shows zero-order correlations between all measures (and Age).

**Table 1:** Descriptives.

Predictor	Mean
*N*	208 (97 female)
NumLine^1^	14.20 (0.39)
NumComp^2^	1738 (29)
DotComp^2^	1721 (31)
Ravens^3^	25.6 (0.3)
NumOrd^2^	4945 (127)
ObjMatch^2^	4979 (93)
DotEst^1^	8.46 (0.20)
Counting^2^	3665 (62)
VisAud^2^	1898 (28)
Reading^3^	49.9 (1.8)
StimResp^2^	970 (12)
Age	7.06 (0.03)


*Values in parentheses are SE of the mean unless indicated otherwise. Superscripts refer to scoring metrics: ^*1*^percent absolute error, ^*2*^composite measure of ER and RT, and ^*3*^number correct. See Materials and Methods for further details on these scoring metrics.*

### Unique Predictors of Number-Line Estimation

**FIGURE 2:**
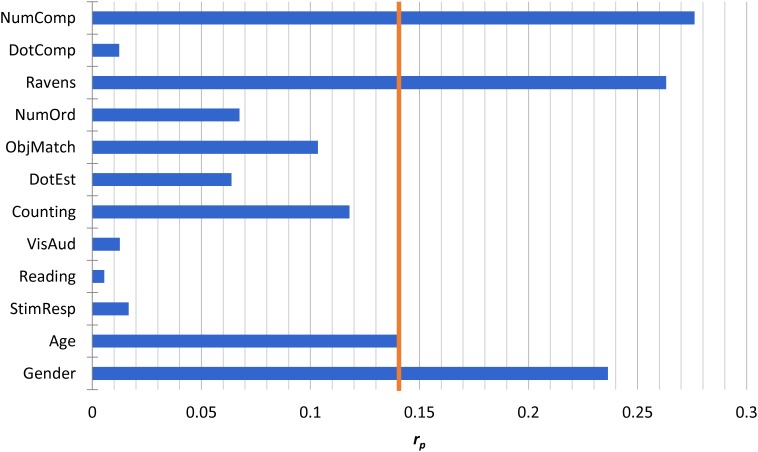
Unique NumLine predictors. The figure shows partial-*r* values from the initial (full) model (see Table 2) predicting NumLine performance. The vertical line indicates the partial-*r* value corresponding to *p* = 0.05.

We first entered all numerical measures, all non-numerical measures, and a dummy variable for gender (0 = male, 1 = female) into a regression model to predict NumLine performance. Age was also included as a control measure. Table 2 shows results of the initial model, and Figure 2 visualizes relative partial correlation coefficients taken from the multiple-regression model. Results of the initial model show that only NumComp, Ravens, Gender, and Age explain unique variance in NumLine performance.

**Table 2:** Initial multiple regression model.

DV: NumLine					

Predictor	*b*	*se*	*t*	*p*	*r_p_*
NumComp	4.9E – 3	1.1E – 3	4.01	8.6E – 5	0.276
DotComp	–1.6E – 4	9.6E – 4	–0.17	0.864	–0.012
Ravens	0.35	0.09	3.81	1.9E – 4	0.263
NumOrd	2.0E – 4	2.1E – 4	0.94	0.346	0.067
ObjMatch	5.2E – 4	3.6E – 4	1.45	0.148	0.103
DotEst	–11.45	12.83	–0.89	0.373	0.064
Counting	–8.3E – 4	5.0E – 4	–1.66	0.099	–0.118
VisAud	1.8E – 4	1.0E – 3	0.18	0.861	0.013
Reading	–1.1E – 3	0.01	–0.08	0.940	–0.005
StimResp	–5.6E – 4	2.4E – 3	–0.23	0.817	–0.017
Age	1.57	0.79	1.97	0.050	0.140
Gender	–2.45	0.72	–3.40	8.2E – 4	0.236


*Overall adjusted *R*^*2*^ = 0.276, numerator *df* = 1 for each predictor. Error (denominator) *df* = 195. *r_*p*_* = partial-*r* value.*

We next aimed to identify the most parsimonious model possible by removing predictors that failed to predict unique variance in NumLine performance, removing predictors with the lowest *p*-values in a step-wise fashion until all predictors were significant at *p* < 0.05. Table 3 shows the progression of model reduction. In the process of model reduction, all predictors were removed with the exception of NumComp, Ravens, and Gender. Because of significant theoretical interest in the DotComp task, we decided to retain it in the final model (shown in Table 4) despite its not predicting unique variance in NumLine performance (indeed, it would have been the second predictor omitted in the process of model reduction). Age was also retained as an important control variable despite not predicting unique variance in NumLine performance.

**Table 3:** Progression of model reduction.

Step	Predictor removed	*p*-value of removed predictor	Adjusted *R*^2^ after predictor removed	Change in adjusted *R*^2^ from initial model
Initial	–	–	0.27609	–
1	Reading	0.940	0.27976	+0.00367
2	VisAud	0.869	0.28332	+0.00723
3	StimResp	0.842	0.28680	+0.01070
4	DotEst	0.369	0.28747	+0.01138
5	NumOrd	0.325	0.28757	+0.01148
6	ObjMatch	0.149	0.28369	+0.00760
7	Counting	0.193	0.28118	+0.00509


*DotComp would have been the second predictor omitted (*p* = 0.870 after reading was removed) but was retained due to significant theoretical interest in this task.*

**Table 4:** Final model details.

DV: NumLine					

Predictor	*b*	*se*	*t*	*p*	*r_p_*
NumComp	4.1E – 3	8.9E – 4	4.60	7.5E – 6	0.308
DotComp	3.7E – 4	8.5E – 4	0.44	0.664	0.031
Ravens	0.36	0.08	4.30	2.6E – 5	0.290
Age	1.27	0.76	1.67	0.096	0.117
Gender	–2.86	0.68	–4.22	3.7E – 5	–0.285


*Overall adjusted *R*^*2*^ = 0.281, numerator *df* = 1 for each predictor. Error (denominator) *df* = 202. *r_*p*_* = partial-*r* value.*

### Modulation by Gender

In this section, we assessed whether the relations between the predictors of interest retained in the final model (NumComp, DotComp, and Ravens) and NumLine were modulated by (interacted with) gender. To do so, we ran a model predicting NumLine in which we interacted NumComp, DotComp, and Ravens with gender. Results (shown in Table 5) demonstrate a significant NumComp x Gender interaction (*p* = 0.023). Results did not show a significant interaction with gender for either DotComp or Ravens (both *p*s > 0.45).

**Table 5:** Gender interaction model.

DV: NumLine					

Predictor	*b*	*se*	*t*	*p*	*r_p_*
NumComp	5.6E – 3	1.1E – 3	4.97	1.5E – 6	0.332
DotComp	3.4E – 4	1.1E – 3	0.32	0.748	0.022
Ravens	0.34	0.12	2.90	0.004	0.202
Age	1.27	0.76	1.67	0.096	0.112
Gender	–13.01	6.06	–2.15	0.033	–0.150
NumComp ^∗^ Gender	–4.1E – 3	1.83E – 3	–2.24	0.026	–0.157
DotComp ^∗^ Gender	–3.5E – 4	0.001	–0.20	0.844	–0.014
Ravens ^∗^ Gender	0.09	0.17	0.57	0.570	0.040


*Overall adjusted *R*^*2*^ = 0.321, numerator *df* = 1 for each predictor. Error (denominator) *df* = 199. *r_*p*_* = partial-*r* value.*

**FIGURE 3:**
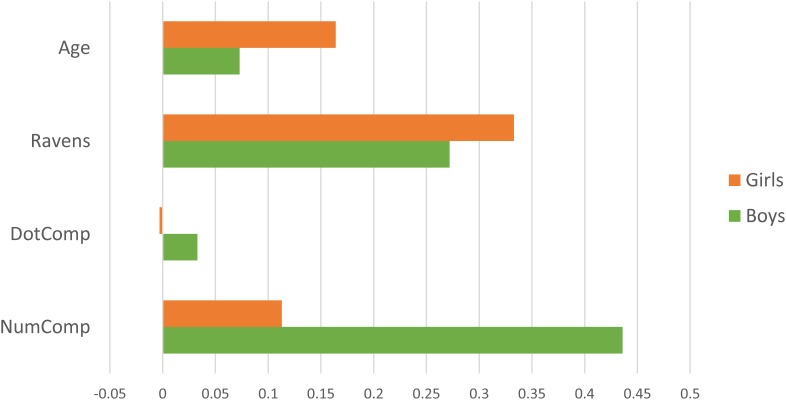
Final NumLine model predictors by gender. The figure shows partial-*r* values predicting NumLine, plotted separately for girls (orange) and boys (green). The partial-*r* values that correspond to *p* = 0.05 is partial-*r* = 0.185 for boys (*N* = 111) and partial-*r* = 0.200 for girls (*N* = 97).

To decompose the significant NumComp x Gender interaction, we next ran multiple-regression models predicting NumLine from NumComp, DotComp, and Ravens, separately by gender. Results (plotted in Figure 3 and shown in Table 6) show that while NumComp was the strongest predictor of NumLine for boys, it did not predict unique NumLine variance for girls. Note that Ravens was a significant predictor for both boys and girls; DotComp was not significant for either.

**Table 6:** Separate models by gender.

DV: NumLine						

	Predictor	*b*	*se*	*t*	*p*	*r_p_*
Boys	NumComp	5.7E – 3	1.1E – 3	4.99	2.4E – 6	0.436
	DotComp	3.6E – 4	1.1E – 3	0.34	0.735	0.033
	Ravens	0.34	0.116	2.91	0.004	0.272
	Age	0.73	0.98	0.75	0.456	0.073
Girls	NumComp	1.6E – 3	1.4E – 3	1.09	0.279	0.113
	DotComp	–4.8E – 5	1.4E – 3	–0.03	0.974	–0.003
	Ravens	0.41	0.122	3.39	0.001	0.333
	Age	1.92	1.21	1.60	0.113	0.164


*Boys: Overall adjusted *R*^*2*^ = 0.302, numerator *df* = 1 for each predictor. Error (denominator) *df* = 106. Girls: Overall adjusted *R*^*2*^ = 0.162, numerator *df* = 1 for each predictor. Error (denominator) *df* = 92. *r_*p*_* = partial-*r* value.*

## Discussion

Across a range of ages and contexts, children’s ability to map numbers into a spatial context has been shown to be a powerful predictor of math performance (Siegler and Booth, 2004; Booth and Siegler, 2008; Sasanguie et al., 2013; Lyons et al., 2014; Friso-van den Bos et al., 2015; Schneider et al., 2018). The goal of the present work was to assess which numerical and non-numerical cognitive abilities predict unique variance in 0–100 number-line estimation ability in first graders. Results indicated that symbolic number processing, but not non-symbolic number processing, predicted unique variance in number-line estimation ability. Moreover, within the realm of symbolic number processing, it was numerical magnitude comparison that was predictive of unique number-line variance, while other symbolic measures, like numeral ordering, did not predict unique variance. The number-line task has been conceptualized as indexing children’s underlying representation of numerical magnitude (Laski and Siegler, 2007; Booth and Siegler, 2008); the present work suggests that number-line estimation is indeed best predicted by measures of numerical magnitude. Crucially, however, our work here indicates that this interpretation is specific to measures of *symbolic* magnitude representation. Furthermore, results showed that non-verbal reasoning ability also predicted unique variance in number-line estimation, suggesting a role for non-numeric, domain-general cognitive abilities in number-line performance. Interestingly, we also found that the relationship between number-line estimation ability and numeral comparison ability was modulated by gender such that numeral comparison was predictive for boys, but not girls. Our results help clarify the nature of the numerical magnitude representations indexed by number-line estimation tasks in early grade-school. Moreover, as number-lines are a ubiquitous visualization device found in early mathematics classrooms, our results may also point to practical implications for the kinds of basic abilities that permit children to get the most out of this common pedagogical tool.

Recent work has demonstrated that symbolic and non-symbolic representations of quantity are distinct in both adults and young children (Verguts et al., 2005; Carey et al., 2017; Lyons et al., 2012, 2015, 2018). Siegler and colleagues have argued that the number-line task assesses underlying magnitude representations (Laski and Siegler, 2007; Booth and Siegler, 2008), but until this point it hasn’t been explicitly tested whether the theorized underlying magnitude is symbolic or non-symbolic. Our findings show it is *symbolic* magnitude comparison that predicts unique variance in number-line estimation ability, whereas approximate magnitude comparison does not. If the number-line task reflected the representational precision of non-symbolic quantities (i.e., the width or narrowness of non-symbolic tuning curves), the NumLine task should have shown a strong relation with children’s ability to distinguish between two non-symbolic magnitudes (indexed here via the DotComp task). However, our results indicated this was not the case. Instead, we found that number-line estimation precision is more closely associated with children’s ability to judge the relative magnitudes represented by number *symbols* (indexed here via the NumComp task). Our results thus clarify an important point with respect to a prominent view of what is indexed by number-line estimation tasks (Siegler and Braithwaite, 2017). Namely, while our results are broadly consistent with the view that number-line tasks primarily index relative magnitude processing (Laski and Siegler, 2007; Booth and Siegler, 2008), here we add the important caveat that the operative notion of magnitude is primarily the *symbolic* aspect of numerical magnitude.

An important question that follows is what exactly is meant by symbolic numerical magnitude (at least in the present context)? As noted above, recent work has indicated that the meaning of number symbols is likely relatively distinct from approximate magnitudes (Verguts et al., 2005; Lyons et al., 2012, 2015, 2018; Carey et al., 2017), and our results here are broadly consistent with this. In response, some have proposed that number symbols are primarily associative in nature, drawing much of their meaning from associations (such as relative order – ‘What comes next?’) with other number symbols (Nieder, 2009; Núñez, 2017; Lyons and Beilock, 2018). However, in the current context of understanding number-line estimation, this associative aspect of number symbols does not appear to be the critical factor either, as we failed to find that performance on the symbolic number ordering task (NumOrd) predicts unique NumLine variance. An alternative hypothesis proposed by Verguts et al. (2005); see also Roggeman et al. (2007) is that exact representation of numbers (as is thought to be the case with number symbols) operates via ‘place coding.’ Numbers are represented with equal precision regardless of numerical magnitude and indexed based on their relative position on a putative internal mental number-line. Perhaps, most intriguingly here, this mental number-line is typically conceptualized in an explicitly visuospatial manner. If it were the case that, rather than just serving as a useful metaphor, children may actually represent numerical magnitudes by placing numbers along a mental line. In such a framework, the precision with which a given quantity is placed on this *mental* line should translate directly to the precision with which it is placed on an *external* line, as in number-line tasks. It may be that first graders rely on this place-based coding to represent symbolic quantities. Hence, this place-based coding may underlie both their ability to compare symbolic magnitudes and generate number-line estimates, as indicated by the strong unique relation between these two tasks we see here.

Here we also found that non-verbal reasoning ability (Ravens) predicted unique variance in number-line performance, suggesting a role for non-numerical cognitive ability in number-line estimation. Previous work on number-line estimation has found individual differences in strategy use (Booth and Siegler, 2008; Slusser et al., 2013; Dackermann et al., 2015; Peeters et al., 2016; van’t Noordende et al., 2016), so a potential interpretation of this relation is that stronger non-verbal reasoning skills may allowing children to select more effective strategies. One practical implication is that future work using the number-line estimation task should take care to control for non-verbal reasoning ability in order to ensure that any claims made about the number-line task are not unknowingly driven by its relation with non-verbal reasoning. From a theoretical perspective, the finding that both numerical magnitude representation and non-verbal reasoning ability each predict unique variance in number-line estimation suggests that both types of ability (numerical and non-numerical) work in conjunction to support effective number-line estimation.

Another result of potential interest here is that the relation between symbolic magnitude comparison and number-line estimation ability was modulated by gender: while this relation obtained for boys (*r*_p_ *=* 0.436), it did not for girls (*r*_p_ = 0.164). Given the preceding discussion, one question is thus why girls did not show a significant relation between NumComp and NumLine performance. Boys consistently show a higher spatial skills on average than girls (Voyer et al., 1995; Kimura, 1999; Terlecki and Newcombe, 2005; Feng et al., 2007). Therefore, one possibility is that, owing to lower general spatial skills, girls are less likely on average than boys to develop an explicitly visuospatial place-coding representation of numerical magnitude, or girls may do so later in development than boys. Consistent with this notion, previous work has found an advantage for boys in number-line estimation (Hutchison et al., 2018). For boys, the number-line task is already cognitively aligned to the spatial manner in which they represent numbers. By contrast, if girls do not primarily represent numbers spatially, there will be an additional cost of translating from a non-spatial representation in order to plot a number in space. Moreover, this putative difference in number representation would also explain the lack of a unique relation between numerical magnitude representation (as indexed by the NumComp task) and number-line performance among girls. If girls do not represent numerical magnitudes spatially, then the ability that allows them to compare symbolic magnitudes would not relate to the ability to plot numbers on a line.

While the idea that boys and girls may vary in the extent to which their representations of numerical magnitudes are spatial in nature is admittedly a *post hoc* interpretation of our results, it does generate some useful hypotheses that may guide future work. First, it suggests that boys’ performance on a number-line task would be harmed more by visuospatial load or by changing the format of the number line (from horizontal to vertical, for instance) than girls’ performance (controlling for general spatial ability). Second, it may be the case that differences in general spatial ability may explain the gender difference in performance on number-line tasks and the simultaneous absence of gender differences on less explicitly spatial measures of numeracy (Hutchison et al., 2018). Moreover, differences in the extent to which representations of numerical magnitude are spatial may also have an impact on how well children learn about numbers and math from spatial pedagogical strategies (discussed below). Finally, it should be noted that we did not find a gender interaction for the Ravens task, suggesting that non-numerical cognitive abilities – regardless of how symbolic magnitudes are being represented – play a similar role for boys and girls.

In addition to informing theories of number-line estimation and informing debates on broader numerical development, we note that the present work has potential implications for educational settings. Number-lines of course arise not just in the context of the eponymous cognitive task, but they are a common pedagogical tool found in early grade-school classrooms used to promote development of numerical understanding. While experimental work would need to be done to lend greater support to this idea, our work suggests that working to promote children’s understanding of *symbolic*, rather than non-symbolic, numerical magnitudes may help children get more out of number-lines as a pedagogical tools. Importantly, however, this may be qualified by gender, applying more strongly to male than female children, on average. Finally, the finding that non-verbal reasoning ability predicts number-line estimation ability (regardless of gender) also suggests that children with lower non-numerical reasoning skills may require additional support when using number-lines as pedagogical tools.

Finally, it is important to note the limitations of the present study. First, this study deals with just one number-line range (0–100). This was done for the practical reason that the majority of children in this age range are familiar with two-digit numbers, but not all may be comfortable with three-digit numbers, so using the range 0–100 is perhaps best suited for the majority of students at this age. Second, the data reported here focused on a single age-range (first graders). This was because number-line estimation ability on 0–100 tasks is still developing for children of this age. As such, focusing on this age range and task presented an opportunity to investigate factors that may affect the development of number-line estimation ability. Furthermore, it should be noted that previous work on number-line estimation has shown that findings from different age groups and different number-line ranges have generalized well to one another (e.g., Siegler and Opfer, 2003; Siegler and Booth, 2004; Booth and Siegler, 2008; Siegler and Ramani, 2009). One might argue a third potential limitation is that our findings were biased to show effects of symbolic number comparison over non-symbolic number comparison because the target magnitudes in the number-line task were presented as symbols rather than dot arrays. However, we controlled for several other symbolic measures, including ordering and number-naming, and, it should be noted, none of those predicted unique variance. This suggests that the effect of numeral comparison we found is not merely driven by the fact that it shares a format with the target magnitude.

## Conclusion

Our work shows that unique variance in number-line estimation ability is explained by individual differences in symbolic magnitude processing and non-verbal reasoning ability, but not approximate magnitude processing. This finding refines theories of number-line estimation by clarifying that the representations of numerical magnitude tapped by the number-line task appears to be largely symbolic in nature rather than reflecting the degree of representational precision of approximate tuning curves. However, the relation between performance on a symbolic magnitude task and number-line estimation was found to be stronger for boys than girls, potentially due to differences in the degree to which number representations are spatial in nature among boys and girls. This work suggests that promoting children’s understanding of symbolic, rather than non-symbolic, numerical magnitudes may help children learn better from number-lines in the classroom and that future research should treat number-line estimation tasks as reflecting underlying representations of symbolic magnitude.

## Author Contributions

Data for the study came from a preexisting dataset. All authors contributed to the conception of the study and writing of the manuscript. RD completed the data analysis.

## Conflict of Interest Statement

The authors declare that the research was conducted in the absence of any commercial or financial relationships that could be construed as a potential conflict of interest.
